# A change to *Experimental Physiology*’s statistics policy

**DOI:** 10.1113/EP091248

**Published:** 2023-04-20

**Authors:** Damian Bailey, George Rose, Alex Stewart

**Affiliations:** ^1^ University of South Wales Neurovascular Research Laboratory Pontypridd United Kingdom; ^2^ University of South Wales Neurovascular Research Laboratory South Wales United Kingdom; ^3^ The Physiological Society Unknown London United Kingdom

Our readership will probably be aware of the ‘replication crisis’ in scientific research. This phrase was catapulted into the vocabulary of the mainstream scientific community in 2012 (Pashler & Harris, 2012). In essence, it described a phenomenon, apparent across a range of research designs and fields, that many, if not most, claimed research findings were simply not reproducible (Ioannidis, 2005).

This lack of reproducibility is evident in the life sciences and casts doubt upon the generalizability of research findings. One of the largest meta‐analyses exploring this phenomenon concluded that low levels of reproducibility, potentially affecting more than half of all preclinical biomedical research, were delaying life‐saving therapies, increasing pressure on research budgets and raising costs of drug development, with US$28 billion a year spent needlessly on preclinical research in the USA alone (Simcoe, 2015).

Multiple explanations for this crisis have been proffered, although it is widely accepted that poor standards of data reporting and statistical rigour have been a major factor (Gandevia, 2021). Back in 2012, the journals of The Physiological Society sought to help improve these standards. To this end, *The Journal of Physiology* jointly published a collection of guidelines on best practices in statistical reporting, in conjunction with the British Pharmacological Society (Drummond et al., 2011). The hope was that by pointing authors towards relevant information, the quality of data reporting and statistical methods published in our journals would improve automatically.

Unfortunately, this hope did not become reality. A cross‐sectional analysis found no evidence that reporting practices in *The Journal of Physiology* were improved within a 4‐year time frame following the publication of this editorial advice (Diong et al., 2018). In the vast majority of papers, SEMs were used inappropriately to summarize data variability, in >90%, exact *P*‐values were not used for primary analyses and post‐hoc tests, raw data were scarcely plotted, and often *P*‐values ≥0.05 were interpreted as trends or statistically significant (Diong et al., 2018).

As a result, it was decided that instead of encouraging best data and statistical practices, our journals should mandate them. In light of this, *The Journal of Physiology* introduced its new statistics policy in 2019 (Forsythe et al., 2019). This policy outlines a number of requirements with which authors must comply before acceptance. These requirements include, but are not limited to: (1) providing a statistical summary document for revised research articles, which is included as Supporting Information in the published paper; (2) using SD, not SEM (unless clearly justified and exempted); (3) providing mean and SD values in the text and statistical summary document; (4) providing precise *P‐*values; and (5) providing all underlying data, either within the paper or as Supporting Information.

*Experimental Physiology* adopted this statistics policy 18 months later. We recently consulted our authorship and Editorial Board for feedback on it and, as a result, will be making an important modification, nearly 2 years after its introduction. Specifically, *Experimental Physiology* will no longer require authors to complete a statistical summary document. Our author base (88% of respondents) and editors were overwhelmingly supportive of the need for a strict policy and the vast majority of the elements in the existing policy. However, there was a general consensus that the ‘duplication’ of work required to complete and check this document was an unnecessary and excessive time burden.

As a journal, we are committed to listening to the feedback of the community we serve. Guided by the sentiments and facts, this change will come into force with immediate effect. Authors must still meet the requirements of our statistics policy within their manuscript, meaning that the level of statistical rigour demanded is not diminished. We are also committed to reducing publication biases through other means, including data‐sharing statements and use of data file repositories, and our Registered Reports article format, in which the methods and analyses of a proposed study are peer reviewed before formal experimentation. By continuing to adopt and modify best practices, we hope to continue to serve and shape our research community and improve the discipline of physiology.

## AUTHOR CONTRIBUTIONS

All authors have approved the final version of the manuscript and agree to be accountable for all aspects of the work. All persons designated as authors qualify for authorship, and all those who qualify for authorship are listed.

## CONFLICT OF INTEREST

None declared.

## FUNDING INFORMATION

None.
